# Differential protein structural disturbances and suppression of assembly partners produced by nonsense GABRG2 epilepsy mutations: implications for disease phenotypic heterogeneity

**DOI:** 10.1038/srep35294

**Published:** 2016-10-20

**Authors:** Juexin Wang, Dingding Shen, Geqing Xia, Wangzhen Shen, Robert L. Macdonald, Dong Xu, Jing-Qiong Kang

**Affiliations:** 1 Department of Computer Science and Christopher S. Bond Life Sciences Center, University of Missouri, Columbia, MO, 65211, USA; 2 Graduate Program of Neuroscience, Vanderbilt University, Nashville, TN 37212, USA; 3 Department of Neurology, Vanderbilt University Medical Center, Nashville, TN 37212, USA; 4 Department of Molecular Physiology and Biophysics, School of Medicine, Vanderbilt University, Nashville, TN 37212, USA; 5 Department of Pharmacology, School of Medicine, Vanderbilt University, Nashville, TN 37212, USA.; 6 Vanderbilt Brain Institute, Vanderbilt University, Nashville, TN 37212, USA.

## Abstract

Mutations in GABA_A_ receptor subunit genes are frequently associated with epilepsy, and nonsense mutations in *GABRG2* are associated with several epilepsy syndromes including childhood absence epilepsy, generalized tonic clonic seizures and the epileptic encephalopathy, Dravet syndrome. The molecular basis for the phenotypic heterogeneity of mutations is unclear. Here we focused on three nonsense mutations in *GABRG2* (*GABRG2*(R136*), *GABRG2*(Q390*) and *GABRG2*(W429*)) associated with epilepsies of different severities. Structural modeling and structure-based analysis indicated that the surface of the wild-type γ2 subunit was naturally hydrophobic, which is suitable to be buried in the cell membrane. Different mutant γ2 subunits had different stabilities and different interactions with their wild-type subunit binding partners because they adopted different conformations and had different surface hydrophobicities and different tendency to dimerize. We utilized flow cytometry and biochemical approaches in combination with lifted whole cell patch-clamp recordings. We demonstrated that the truncated subunits had no to minimal surface expression and unchanged or reduced surface expression of wild-type partnering subunits. The amplitudes of GABA-evoked currents from the mutant α1β2γ2(R136*), α1β2γ2(Q390*) and α1β2γ2(W429*) receptors were reduced compared to the currents from α1β2γ2 receptors but with differentially reduced levels. This thus suggests differential protein structure disturbances are correlated with disease severity.

Mutations in *GABRG2* are associated with epilepsies of varying severities. However, the basis for the mutant γ2 subunits structure and the correlation between structural disturbances and disease phenotypes has not been reported. We have demonstrated that nonsense *GABRG2* mutations result in loss-of-function but different nonsense mutations are associated with epilepsy phenotypes with different severities. Thus, understanding the structural alterations of mutant γ2 subunits may provide novel insights into epilepsy phenotypic heterogeneity. *GABRG2*(136*) is a mutation associated with febrile seizures (FS)^1^, *GABRG2*(Q390*)^2^ is a mutation associated with the severe epilepsy Dravet syndrome, and *GABRG2*(W429*)^3^ is a mutation associated with FS and the moderately severe genetic epilepsy with FS plus (GEFS+). We have demonstrated that protein degradation rate is associated with steady state protein expression of the mutant GABA_A_ receptor γ2 subunit^4^. However, the structural basis for the mutant protein’s stability, and the correlating biochemistry and function of the mutant subunits has not been reported.

Although the GABA_A_ receptor is a major mediator of fast inhibitory neurotransmission in the CNS, and the assembly and current kinetic properties of GABA_A_ receptors have been well characterized, the structure of the receptor is less known. Among GABA_A_ receptor subunits, the first three-dimensional structure of the GABA_A_ receptor β3 homopentamer was resolved by X-ray diffraction and has revealed many architectural details of the homopentamer and its role as a pentamer in channel signal transduction^5^. However, other unsolved subunits of GABA_A_ receptors could also oligomerize and produce different pentamers with various functional roles and their structures remain unknown. In addition, missense mutations and nonsense mutations with truncations of different lengths of these subunits still lack structure-based explanation of their properties. Protein structure prediction provides a powerful tool to infer tertiary structure from protein amino acid sequence^6^. With structural modeling and protein docking, there are already several successes in predicting function-related structural conformational differences between mutant/truncated and wild-type structures^7^,^8^,^9^,^10^,^11^.

In the present study, we characterized the properties of the three FS and epilepsy associated truncated mutant γ2 subunits based on structural modeling. Based on the predicted GABA_A_ receptor subunit structural models and a series of computational analyses, we quantitatively inferred the protein-protein interaction stabilities among these subunits in the complexes. Our computations are mainly rooted in one widely accepted hypothesis on stability of protein complexes: if the predicted binding affinity is higher, then the proposed protein-protein complex is likely more stable and has a higher probability to exist *in vivo*^9^,^12^,^13^. In particular, we have demonstrated that differences in protein stability are due mainly to the differentially accessible surface area (*ASA*) and surface hydrophobicity^14^. ASA is protein surface area accessible to a solvent from solvent probe radius 1.4 Å as calculated by nACCESS^15^. With various protein docking processes, we have determined that different mutant subunits have different interactions with the remaining wild-type partnering subunits, like α1 subunits, and the stabilities of the dimers of different mutant subunits are different.

We have used biochemistry and flow cytometry to further validate the results of protein structural modeling. We have determined total and surface expression of the three mutant γ2 subunits. We have determined the propensity of the mutant subunits to form high molecular mass protein aggregates. With a de-glycosylation study, we have demonstrated the differential glycosylation arrest of the wild-type subunits when co-expressed with the different mutant γ2 subunits and endoplasmic reticulum (ER) retention of the wild-type partnering subunits. With whole-cell patch clamp recordings, we have identified different extents of preservation of wild-type channel function.

## Results

### Wild-type and mutant GABA_A_ receptor γ2 subunits had different surface hydrophobicity scores

**Figure 1:**
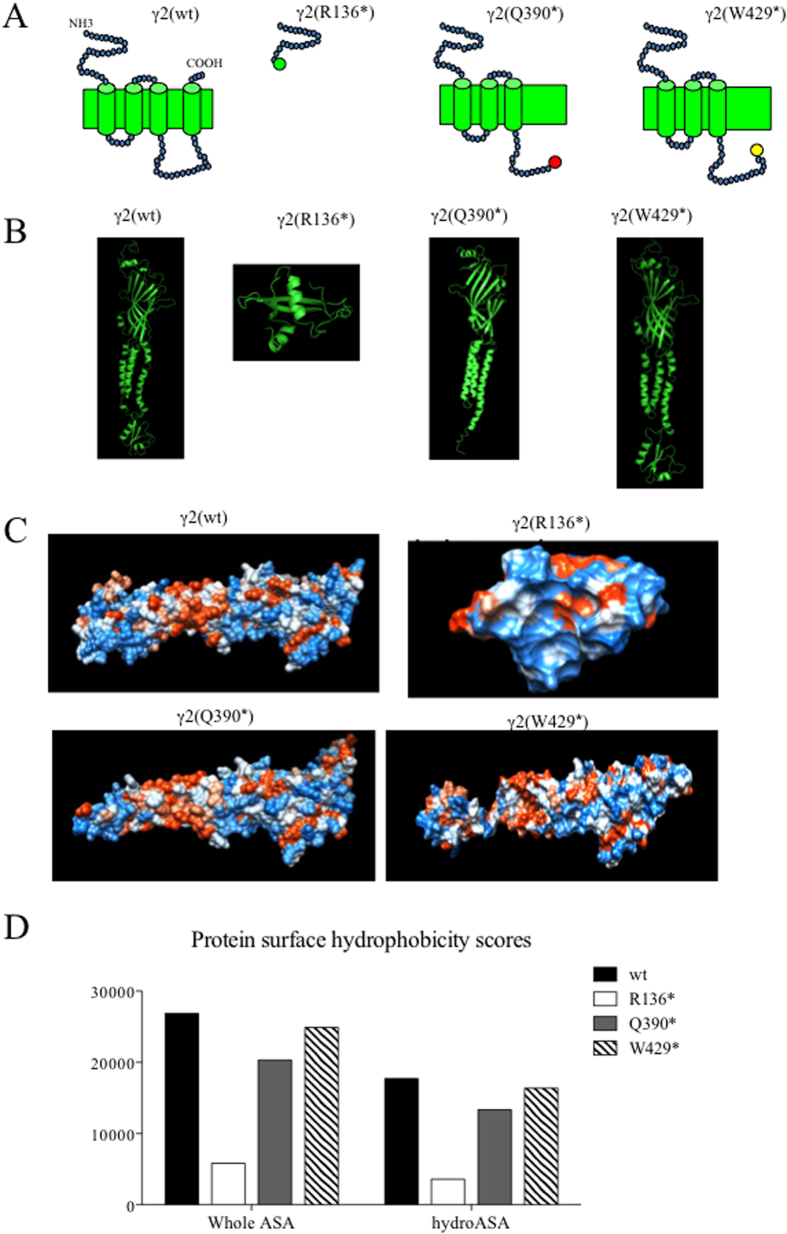
Differential protein surface hydrophobicities of mutant γ2 subunits. (**A**) The schematic illustration of the wild-type γ2 and the mutant γ2(R136*), γ2(Q390*) and γ2(W429*) subunits. (**B**) Predicted protein structural models of the wild-type γ2 and the mutant γ2(R136*), γ2(Q390*) and γ2(W429*) subunits. All structural models were predicted by MUFOLD and presented by PyMOL. (**C**) Predicted protein surface hydrophobicity. Orange stands for hydrophobic residues and blue stands for hydrophilic residues. The protein surfaces were shown by Chimera. (**D**) Histogram showing the whole accessible surface area (whole ASA) and hydrophobicity surface accessible area (hydroASA).

We determined structural alterations of the three mutant γ2 subunits based on protein homology modeling (Fig. 1A). The *GABRG2*(R136*) mutation resulted in a loss of a portion of the N-terminus, all four transmembrane domains and all extracellular and intracellular loops with the only the short upstream N-terminal peptide remaining. The *GABRG2*(Q390*) mutation resulted in the loss of the downstream 78 amino acids in the middle of the intracellular TM3-TM4 loop towards the C-terminus while the *GABRG2*(W429*) mutation resulted in loss of the downstream 39 amino acids in the middle of the TM3-TM4 intracellular loop towards the C-terminus. With MUFOLD, structural homology modeling of wild-type and three mutant γ2 subunits was illustrated (Fig. 1B). It is of note that all the mutant γ2 subunits we presented here are whole proteins including sequences of N-terminus, transmembrane domain to intracellular loop while only part of the γ2(Q390*) protein model was reported in our previous study^7^. The hydrophobicity of the protein surface was presented in Fig. 1C. We measured the whole *ASA* and *hydroASA* of the wild-type and mutant subunits as hydrophobicity scores. At monomer level, compared with the wild-type γ2 subunit (17701.32 for *hydroASA* and 26892.48 for whole *ASA*), the mutant γ2(W429*) subunit had similar areas in *hydroASA* (163641 for *hydroASA*; 24877.64 for whole *ASA*). The mutant γ2(Q390*) subunit had reduced values in whole *ASA* and *hydroASA* (13372.87 *hydroASA*; 20332.57 for whole *ASA*) while the mutant γ2(R136*) subunit protein had the most reduced values in whole *ASA* and *hydroASA* (3580.68 *hydroASA*; 5823.09 for whole *ASA*) and (Fig. 1D, Table 1 and Supplementary Table 1).

### Different mutant γ2 subunits formed homodimers with different stabilities

After obtaining structural models of wild-type γ2 and mutant subunits, the homodimers were obtained by symmetrical docking and template-based docking on the corresponding models individually. Using the same procedures as previously described by Yu^16^ and Tsigelny^17^,^18^, we demonstrated that the γ2 subunit homodimers could adopt three different possible conformations. The first conformation was a non-propagating dimer (head-to-tail), which could be obtained by symmetric docking. The second conformation was a propagating dimer that may propagate to a fibril and this dimer could also be obtained by symmetric docking but with membrane constraints, i.e. choosing symmetric docking head-to-head results in both membrane regions located in the membrane. The third conformation was also a propagating dimer that could propagate to a ring structure. We constructed γ2 subunit dimers by adopting the experimentally resolved homo-pentamer as the template.

**Figure 2:**
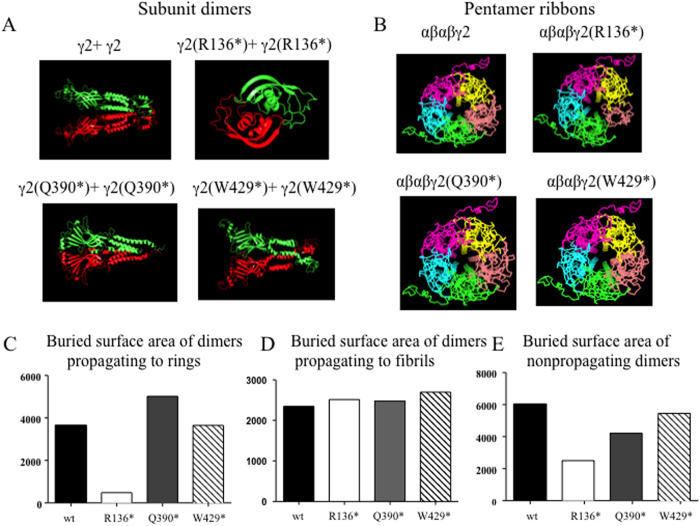
Differential potential mutant γ2 subunit homodimers and oligomers. (**A**) Top docking models for potential mutant γ2 subunit homodimers predicted by SymmDock were shown by PyMOL. In each panel, the two γ2 subunit chains were shown in red and green. (**B**) Alpha-beta-alpha-beta-gamma pentamer ribbons of the wild-type and the mutant γ2 subunit containing receptors. Yellow stands alpha subunit, purple for beta subunit, cyan for alpha subunit, green for beta subunit while red stands for the wild-type or the mutant γ2 subunits. (**C**) The values of buried surface area of the wild-type or the mutant γ2 subunit dimers which could propagate to ring or annular structures. (**D,E**) The values of buried surface area of dimers which could propagate to fibrils (**D**) or nonpropagating dimers (**E**) for the wild-type or the mutant γ2 subunits.

We present the γ2 subunit homodimers predicted by SymmDock were shown by PyMOL. The two γ2 subunit chains were shown in red and green (Fig. 2A). Alpha-beta-alpha-beta-gamma pentamer ribbons of the wild-type and the mutant γ2 subunit containing receptors were also presented (Fig. 2B). We modeled all three possible conformations of mutant γ2 and wild-type γ2 dimers, and calculated the energies which were represented by buried surface values for each of these hypothetically propagating dimers to rings or annular structures (Fig. 2C). A larger buried surface value could represent a larger binding affinity and a more stability of the dimers and a higher likelihood of forming ring or annular structures. The wild-type and mutant γ2(W429*) dimers had similar energy (3647.423 for wt and 3650.25 for W429*) propagating to rings. The mutant γ2(Q390*) dimer had the highest energy (5015.323) while the mutant γ2(R136*) dimer had the lowest energy propagating to rings (482) among all the four γ2 subunit dimers (Fig. 2C). This suggests that γ2(Q390*) subunit dimers are more stable and more likely to form ring or annular structures. We also calculated the energies for propagating fibrils (Fig. 2D) and nonpropagating dimers (Fig. 2E) for the wild-type and mutant γ2 subunits. The energy of the γ2(W429*) dimers propagating to fibrils (2697.394) is similar to the wild-type γ2 subunit dimer (2342.576). The energy of the γ2(Q390*) dimers propagating to fibrils (2475.417) is similar to that of γ2 (R136*) subunit dimers (2513.002) (Fig. 2D). The energies of nonpropagating dimers for the wild-type γ2 subunit (6021.28) were higher than all the mutant γ2 subunits (2513 for γ2 (R136*), 4217.18 for γ2 (Q390*) and 5457.34 for γ2(W429*)) (Fig. 2E).

### Different mutant γ2 subunits had different levels of total protein, and all mutant γ2 subunits were more likely to form dimers and higher oligomers

**Figure 3:**
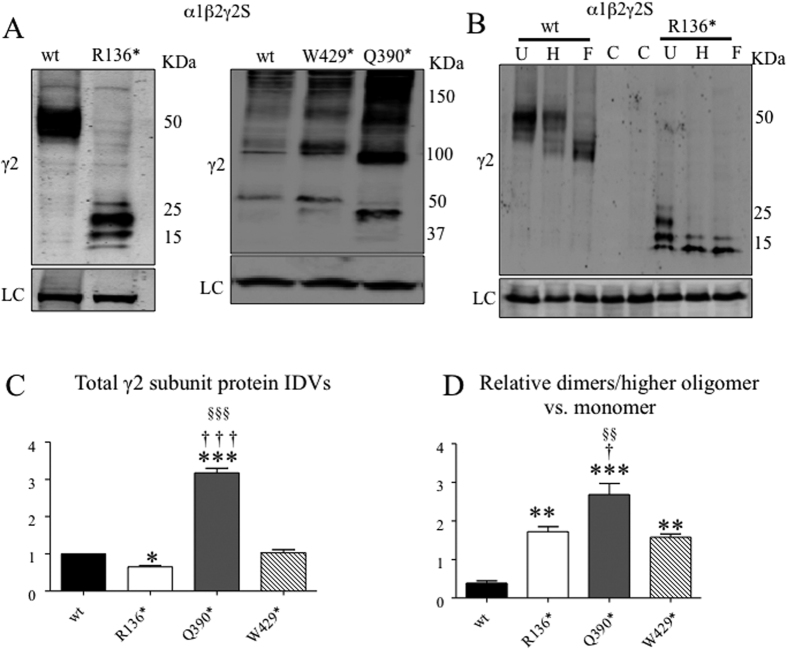
Differential propensity of dimerization/formation of higher oligomers of mutant γ2 subunits. (**A,B**) HEK293T cells were cotransfected with α1, β2 and γ2, γ2(R136*), γ2(Q390*), and γ2(W429*) subunits for 2 days. Total lysates containing γ2 subunits were fractionated by SDS-PAGE and immunoblotted by anti-γ2 subunit antibody. (**A**) The gels for γ2(R136*) subunits were run separately because of the small protein mass of the mutant γ2(R136*) subunits. (**B**) Total lysates from HEK293T cells were cotransfected with α1, β2 and γ2, γ2(R136*) were either untreated or treated with Endo-H (H) or PNGase F (F) and were then fractionated by SDS-PAGE. (**C**) Total mutant subunit band IDVs were normalized to the wild-type γ2 subunits. (**D**) The relative ratio of dimer/high molecular mass complexes normalized to the monomer IDVs. In (**C**,**D**), (*<0.05, **<0.01, vs; ***<0.001 vs wt, ^†^<0.05, ^†††^<0.001 vs R136*, ^§§^<0.01, ^§§§^<0.001 vs W429*). ANOVA with Bonferroni post hoc test was used.

We utilized a biochemical approach to determine expression levels of mutant γ2 subunits and their propensity to dimerize. We co-expressed mutant γ2 subunits with α1 and β2 subunits and determined the total γ2 subunit protein level. We separately analyzed the γ2(R136*) subunit because of its much smaller molecular mass compared with the other mutant subunits. We demonstrated previously that γ2(R136*) subunits migrated in multiple bands, but with reduced amounts, while wild-type γ2 subunits only migrated in one band. In contrast, mutant γ2(390*) and γ2(429*) subunits migrated with multiple bands at higher oligomers and with one band at monomer level with increased protein amount (Fig. 3A). We demonstrated previously that the bands of higher molecular mass in γ2(390*) and γ2(429*) subunits are dimers and higher oligomers^4^,^19^ by pulse chase radio labeling. The dimers as well as the higher oligomers are resistant to detergent as evidenced on SDS gels. It is likely that the multiple bands observed in γ2(R136*) subunits are the different glycosylation forms of the mutant protein dimers as the subunits only migrated in two bands after either Endo H (H) digestion, which removes the ER glycosylation, or PNGase F (F) digestion, which removes all glycans. We observed the identical pattern after H and F digestion, indicating the mutant γ2(R136*) subunits only had ER glycosylation (Fig. 3B). We quantified the total subunit protein amount and demonstrated that the γ2(R136*) subunit had reduced total amount of protein (0.65 ± 0.032, n = 4), γ2(Q390*) subunits had increased total amount of protein (3.175 ± 0.125, n = 4) while the γ2(W429*) subunits (1.025 ± 0.086) had a total amount of protein that was similar to that of wild-type subunit, which was arbitrarily taken as 1 (Fig. 3C). We also determined the relative amount of dimers/higher oligomers compared to monomers in each condition. The dimers/higher oligomers or monomers were normalized to loading control and the ratio of dimers/higher oligomers over monomers was measured. We demonstrated that all three mutant γ2 subunits (1.72 ± 0.13 for R136*, 2.68 ± 0.29 for Q390*, 1.575 ± 0.085 for W429*, n = 4) were more likely to form dimers or higher oligomers compared with wild-type γ2 subunits (0.385 ± 0.06 for wt) (Fig. 3D). Highest steady state amount of higher oligomers and total protein of γ2(Q390*) subunits among all γ2 subunits suggested that the γ2(Q390*) subunits were most stable and were not easily disposed of by the cellular degradation machinery. In summary, compared to wild-type γ2 subunits, γ2(R136*) subunit levels were reduced, γ2(Q390*) subunits had increased total protein, and total γ2(W429*) subunits were unaltered.

### Surface hydrophobicity of γ2 subunits was the highest among GABA_A_ receptor subunits, and the γ2-γ2 dimer was the most stable dimer among all GABA_A_ receptor subunit homodimers

**Figure 4:**
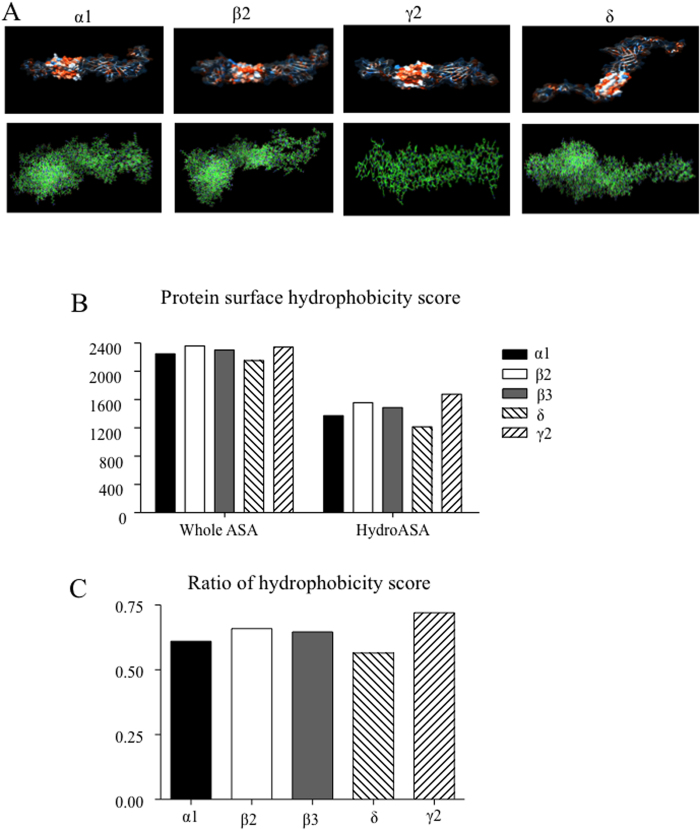
Structural modeling of GABA_A_ receptor subunits and their hydrophobicities. (**A**) Natural hydrophobic surface (upper panel) and atom (lower panel) presentation of GABRA1, GABRB2, GABRG2, and GABRD (from left to right). Hydrophobicity of the residues was presented by different colors. Orange represents hydrophobic residues and blue hydrophilic residues. The transmembrane domain was presented in solid, while other parts were transparent. These figures are presented by Chimera. (**B**) Surface hydrophobicity score of GABA_A_ receptor subunits. (**C**) The ratio of surface hydrophobicity score (*hydroASA/whole ASA*) was plotted.

We previously demonstrated that wild-type γ2 subunits also have a tendency to dimerize when there is no partnering subunit^19^. We modeled wild-type γ2 subunits and compared them with other wild-type subunits including α1, β2 and δ subunits (Fig. 4A). We demonstrated that the hydrophobicity score and the ratio of *hydroASA* over the whole *ASA* of the γ2 subunits were the highest among all the GABA_A_ receptor subunits (Fig. 4B,C and Supplementary Table 2). For all structural models, α, β2, β3, γ2, and δ subunits were treated as monomers, and the homodimers were obtained by symmetric docking on these corresponding models individually. The binding affinities were predicted by these quantitative criteria listed in Supplementary Table 3 Binding affinities obtained from *Hydrophobic Buried Surface Area*, *EmpiricalValue*, *Choi’s dG_est* and *dG_separated/dSASAx100* could explain the phenomenon that wild-type γ2 dimers had the highest binding affinity among all wild-type GABA_A_ receptor subunit dimers, even larger than α1, β3, and δ subunit dimers. *Buried Area ASA* of the wild-type γ2 dimer was a little smaller than the wild-type β2 dimer, which is inconsistent with the observation, while *Packstat* failed to explain the protein stability ranking among these dimers. From these results, we concluded that the wild-type γ2 dimer had the largest buried surface area and the largest hydrophobic buried surface area compared with all other wild-type subunit dimers. The large C-terminus in the intracellular region of γ2 dimers may make it the most stable dimer among all the wild-type dimers.

### There was differential interaction of mutant γ2 subunits with wild-type partnering subunits

**Figure 5:**
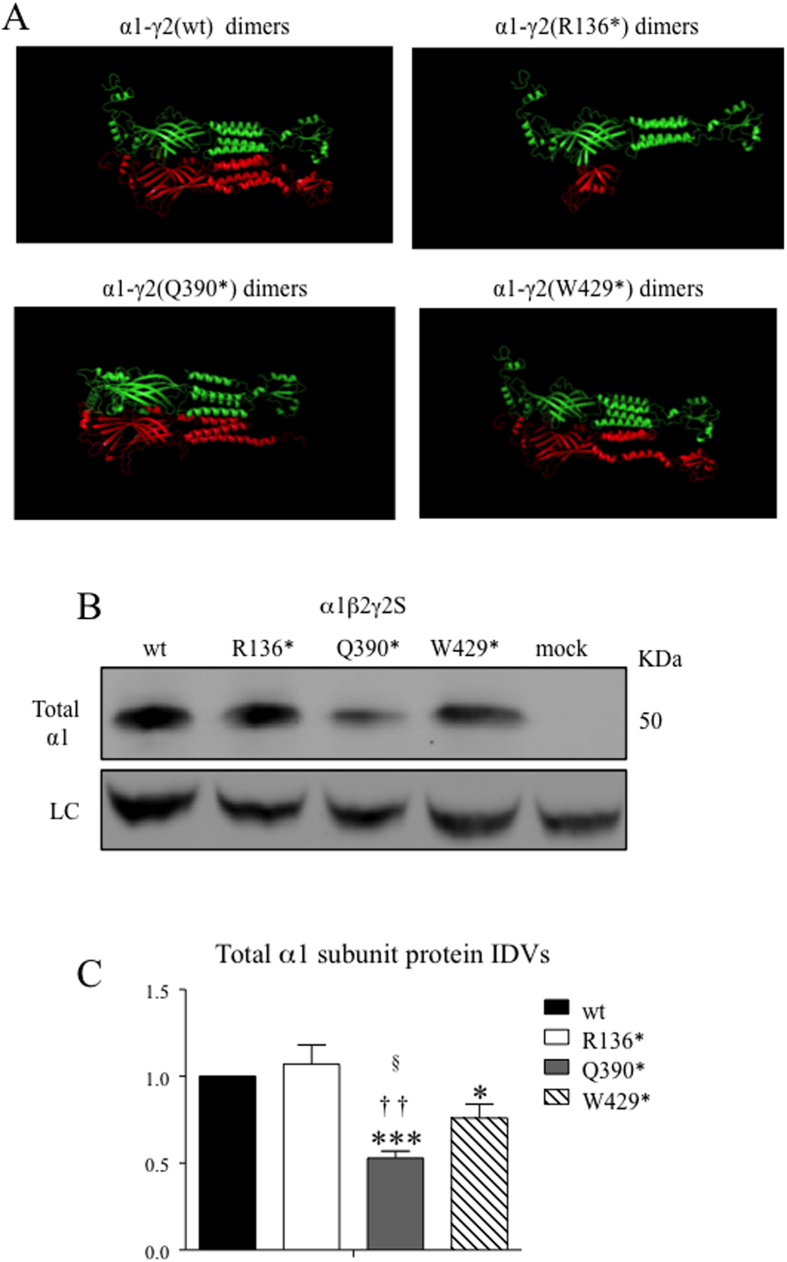
Differential interactions of mutant γ2 subunit with partnering subunits. (**A**) Top docking models of potential complexes between the mutant γ2 subunit (shown in green) and its wild-type partnering α1 subunit (shown in red) predicted by template-based docking were shown by PyMOL. (**B**) Total lysates from HEK293T cells cotransfected with α1, β2 and γ2, γ2(R136*), γ2(Q390*), and γ2(W429*) subunits for 2 days were fractionated by SDS-PAGE and immunoblotted by anti-α1 subunit antibody. The gels were run under the same experimental conditions and were cropped around 50 KDa. The full-length gel for B was presented in Supplementary Figure 1. (**C**) Total mutant subunit band IDVs were normalized to the wild-type γ2 subunits (*<0.05 vs wt; ***<0.001 vs wt) ^††^<0.01 vs R136*, ^§^<0.05 vs W429*). One sample t test and unpaired student t test were used.

Instead of directly symmetrical docking in constructing dimers, mutant β-α-β-α-γ2(R136*), β-α-β-α-γ2(Q390*), β-α-β-α-γ2(W429*) and wild-type β-α-β-α-γ2 pentameric receptors were constructed by template-based docking from the solved β3 homopentamer structure (Fig. 5A). Since the mutant γ2 subunit was the only difference among these pentamers, we only considered the α-γ2 and γ2-β binding affinity variants between the wild-type and mutant pentamers in the template-based docking pentamer. The average of these two binding affinities was assumed to determine the stability of the whole pentamer. The average interface affinities in wild-type α-γ2/γ2-β, mutant α-γ2(R136*)/β-γ2(R136*), mutant α-γ2(Q390*)/β-γ2(Q390X) and mutant α-γ2(W429*)/β-γ2(W429*) subunits illustrate the stability of these pentamers (Supplementary Tables 3 and 4). The binding affinities of all the protein-protein interfaces were detailed in Supplementary Table 4. Binding affinities obtained from buried surface area and empirical score (*Buried Surface Area*, *Hydrophobic Surface Area*, *EmpiricalValue*, *EmpiricalValue’*, and *Choi’s dG_est*) were all consistent with the observation that the mutant pentamer β-α-β-α-γ2(R136*) was less stable than the wild-type pentamer β-α-β-α-γ2, and the wild-type pentamer β-α-β-α-γ2 was less stable than the mutant pentamer β-α-β-α-γ2(Q390*). Only structure-based criterion *dG_separated/dSASAx100* and *Packstat* could not explain the stability rankings.

The structural interpretation from the modeling results was similar to hydrophobicity analysis in mutant subunit dimers. The large truncation in the γ2(R136*) subunit made the binding affinity reduced by the buried surface area shrinking in both interfaces of neighboring α and β subunits. While the truncation in the γ2(Q390*) subunit increased buried surface area in adjacent subunits of the pentamer, the different interactions among the wild-type and mutant γ2 subunits may have different impacts on the biogenesis of wild-type partnering subunits. We have compared the total α1 subunit expression when it was co-expressed with the wild-type β2 subunit and different γ2 subunits. Compared with the α1 subunit co-expressed with the wild-type γ2 subunit, the α1 subunit expression was not changed in the γ2(R136*) subunit condition. In contrast, the α1 subunit expression was reduced when co-expressed with γ2(Q390*) and γ2(W429*) subunits (Fig. 5B). When normalized to the α1 subunit in the wild-type γ2 subunit condition which was arbitrarily taken as 1, the α1 subunit was reduced almost by half when co-expressed with the γ2(Q390*) subunit (0.53 ± 0.04, n = 4) while the α1 subunit was reduced by ~25% when co-expressed with the γ2(W429*) subunit (0.76 ± 0.08, n = 4) (Fig. 5C).

### There was different surface expression of mutant γ2 subunits and their wild-type partnering subunits

**Figure 6:**
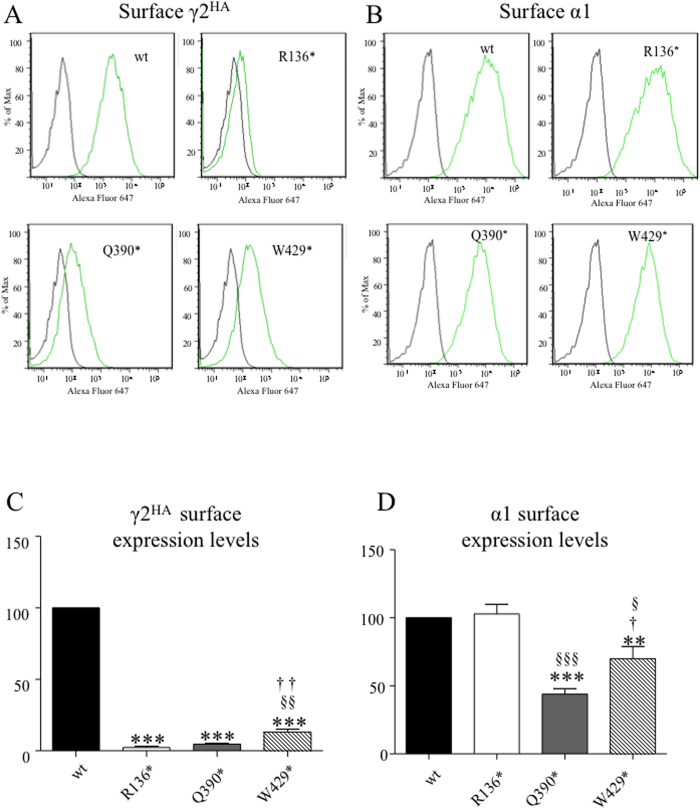
Differential cell surface expression of the mutant γ2 subunits and the partnering α1 subunits. (**A,B**) The flow cytometry histograms depict surface HA levels detected with HA-Alexa 647 (**A**) or α1-Alexa 647 (**B**) With coexpression of γ2^HA^, γ2(R136*)^HA^, γ2(Q390*)^HA^ and γ2(W429*)^HA^ subunits with α1 and β2 subunits in HEK293T cells. (**C**) The relative fluorescence intensities of HA signals from cells expressing the mutant γ2^HA^ subunits were normalized to those from wild-type γ2^HA^ subunits which were arbitrarily taken as 100. (**D**) Relative fluorescence intensities of α1 subunit signals from cells expressing the mutant γ2^HA^ subunits normalized to those from wild-type α1 subunits which were arbitrarily taken as 100. In (**C**,**D**), ***p < 0.001 vs. wt; ^†^p < 0.05, ^††^p < 0.01 vs Q390*, ^§^p < 0.05, ^§§^P < 0.01 vs. W429*. One sample t test and unpaired student t test were used.

Because γ2 subunits have to be co-assembled with α and β subunits to form pentamers before they can traffick to the cell surface and synapses, we co-expressed γ2 subunits with α1 and β2 subunits. We determined the surface expression of the wild-type and mutant γ2 subunits and the wild-type α1 subunit with flow cytometry. When co-expressed with α1 and β2 subunits, surface expression of all three mutant γ2 subunits were reduced substantially (2.25 ± 0.95 for R136*; 4.63 ± 0.69 for Q390*; 13 ± 2.04 for W429*, n = 4) relative to wild-type γ2 subunits (taken as 100) (Fig. 6A,C). However, the surface expression of the γ2(W429*) subunit was higher than that of the γ2(R136*) and γ2(Q390*) subunits.

We then determined surface expression of α1 subunits. The α1 subunit surface expression with co-expression of γ2(R136*) subunits (103 ± 7, n = 4) was not reduced compared with the wild-type. The α1 subunit surface expression was substantially reduced with co-expression of γ2(Q390*) subunits (44 ± 4, n = 4) and reduced to a lesser extent with co-expression of γ2(W429*) subunits (70 ± 9, n = 4) (Fig. 6D).

### Wild-type α1 subunits had different glycosylation and ER retention when co-expressed with mutant γ2 subunits

**Figure 7:**
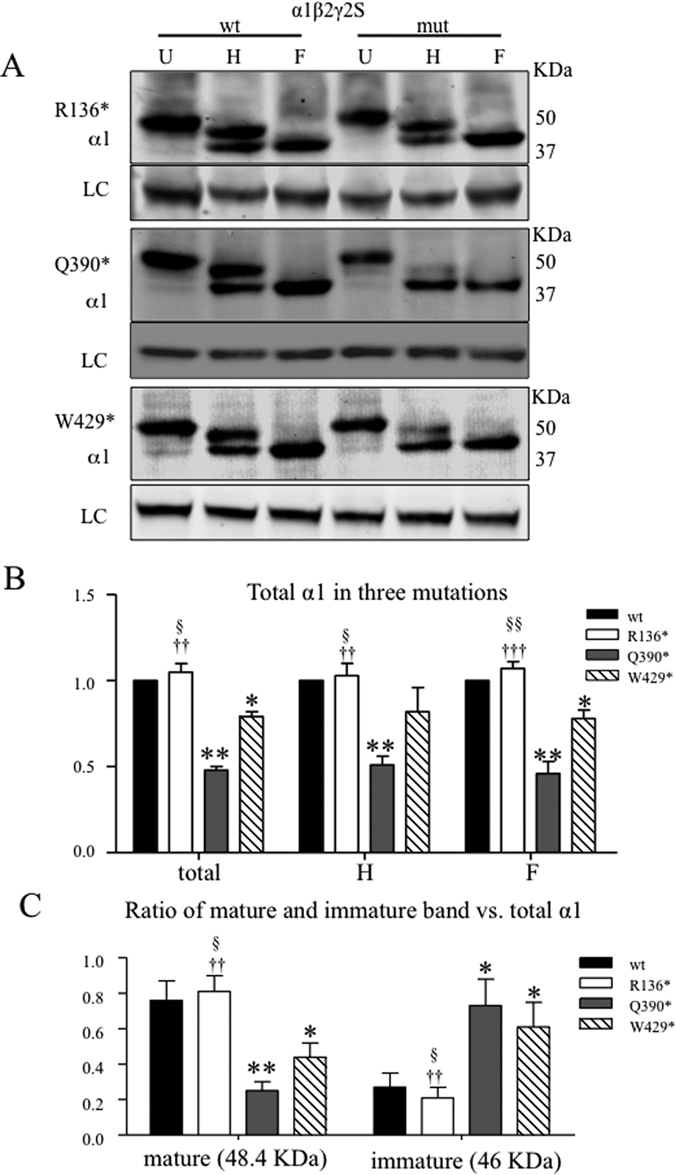
The wild-type partnering α1 subunits had glycosylation arrest when coexpressed with γ2(Q390*) and γ2(W429*) subunits but not with γ2(R136*) subunits. (**A**) HEK293T cells were cotransfected with α1 and β2 subunits and γ2 (wt), γ2(R136*), γ2(Q390*), or γ2(W429*) subunits. Total lysates of these HEK293T cells were undigested (U) or digested with Endo H (H) or PNGase F (F) followed by SDS-PAGE and probed with anti-α1 subunit antibody. After Endo-H digestion, α1 subunits migrated in 48.4 KDa and 46 KDa. The gels were run under the same experimental conditions and were cropped around 50 KDa. The full-length gel for A was presented in Supplementary Figure 1. (**B**) The total α1 subunit protein in U, H and F condition for the wild-type and the mutant α1β2γ2 receptors was quantified and normalized to the wild-type α1 subunit. In H, the IDVs of 48.4 KDa and 46 KDa bands were added. (**C**) The ratios of the mature (48.4 KDa) or the immature (46 KDa) band vs total α1 subunit in untreated condition (U) were plotted. In (**B**,**C)**, *p < 0.01, **p < 0.01 vs wt; ^†^p < 0.05, ^††^p < 0.01 vs Q390*, ^§^p < 0.05, ^§§^P < 0.01 vs W429*. ANOVA with Bonferroni post hoc test was used.

ER retention and ER associated degradation (ERAD) are common pathways for disposal of misfolded mutant proteins. The ERAD quality control pathway is conserved for all the glycoproteins including GABA_A_ receptor subunits^20^. We have demonstrated that both wild-type and mutant GABA_A_ receptor subunits are subject to ERAD. We co-expressed α1 and β2 subunits with wild-type or mutant γ2 subunits in HEK cells, obtained total cell lysates for each transfection condition, and treated them with Endo H or PNGase F followed by analysis with SD-PAGE. With Endo H digestion, the α1 subunit migrated at 48.4 and 46 KDa, and the 48.4 KDa band contained the mature form while the 46 KDa band contained the immature form (Fig. 7A and Supplementary Figure 1), as previously reported^21^. Total α1 subunit levels were not changed with co-expression of mutant γ2(R136*) subunits (1.05 ± 0.05 for U, 1.03 ± 0.07 for H, 1.07 ± 0.04 for F, n = 4); but were reduced with co-expression of either γ2(Q390*) subunits (0.48 ± 0.02 for U, 0.51 ± 0.05 for H, 0.46 ± 0.07 for F, n = 4) or γ2(W429*) subunits (0.79 ± 0.03 for U, 0.82 ± 0.14 for H, 0.78 ± 0.05 for F) (Fig. 7B). The α1 subunit was more reduced with co-expression of γ2(Q390*) subunits than of γ2(W429*) subunits. We then compared the relative ratio of the mature or the immature form to the total α1 subunit protein. The mature form of α1 subunits are trafficked beyond the ER and reach the cell surface while the immature form resides in the ER. There were no differences in the ratios of mature and immature α1 subunit to the total α1 subunit for the γ2(R136*) subunit (0.76 ± 0.11 for mature wt vs 0.81 ± 0.09 for mature R136*; 0.27 ± 0.08 for immature wt vs 0.21 ± 0.06 for immature R136*). However, the ratio of the mature to total α1 subunit was reduced with coexpression of γ2(Q390*) (0.25 ± 0.05 for mature Q390*; and γ2(W429*) (0.44 ± 0.08 for mature γ2(W429*) subunits. In contrast, the ratio of the immature band to the total α1 subunit was increased with co-expression of the two mutant subunits (0.73 ± 0.15 for the immature γ2(Q390*) subunit; 0.61 ± 0.14 for the immature γ2(W429*) subunit) (n = 4) (Fig. 7C). The increased presence of the immature α1 subunit and glycosylation arrest was likely due to the oligomerization of α1 and γ2 subunits and stable interactions between these subunits. Consequently, thee immature subunits would be degraded by ERAD, resulting in decreased surface expression of the α1 subunits and reduced total current.

### Different γ2 mutant subunits co-expressed with α1 and β2 subunits produced receptors with different channel functions

**Figure 8:**
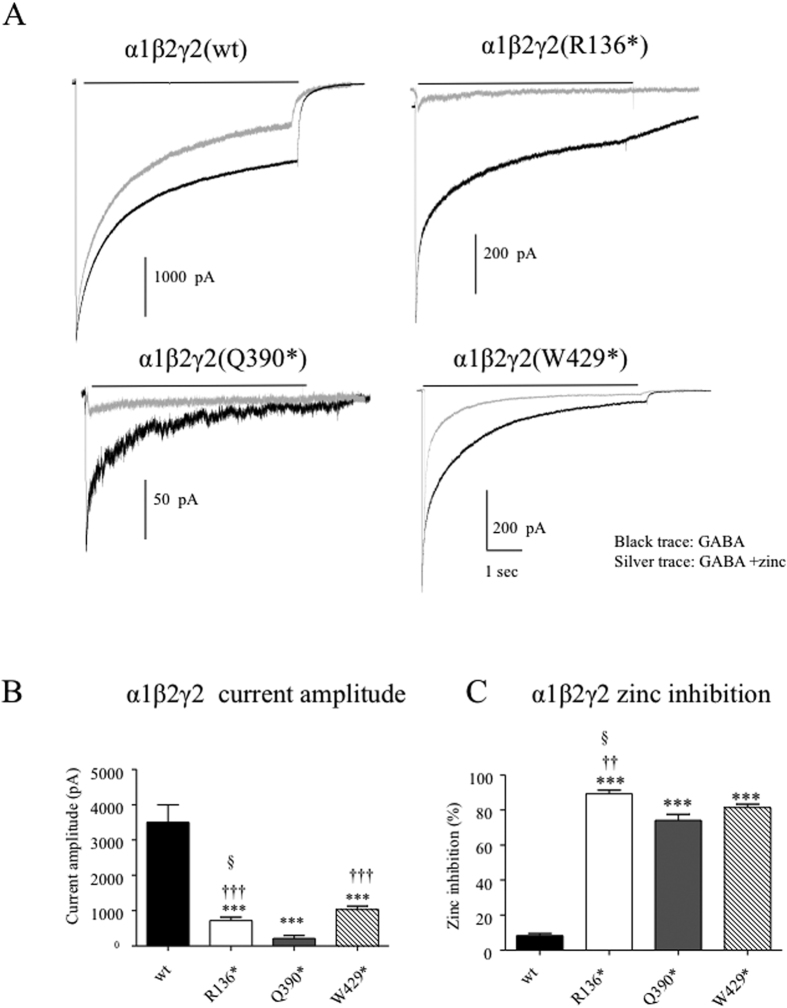
Currents recorded from cells expressing all the mutant γ2 subunits in combination with α1 and β2 subunits had reduced peak current amplitudes and were more sensitive to zinc inhibition. (**A**) GABA_A_ receptor currents were obtained from HEK293T cells co-expressing α1 and β2 subunits with wild-type γ2, mutant γ2(R136*), γ2(Q390*) or γ2(W429*) (1:1:1 cDNA ratio), subunits with application of 1 mM GABA for 6 sec (black trace). (**B**) The amplitudes of GABA_A_ receptor currents from (A) were plotted. Values were mean ± SEM (n = 8–15 patches from 4 different transfections) (***p < 0.001 vs. wt, ^†††^p < 0.001 vs Q390*, ^§^p < 0.05 vs W429*). (**C**) GABA_A_ receptor currents were obtained with 1 mM GABA applied for 6 sec (black trace) and co-application of 1 mM GABA with 10 μM zinc after pre-application of 10 μM zinc (silver traces). The cells were pre-applied with zinc (10 μM) for 6 sec before co-application. The percent reduction of peak amplitude of GABA_A_ receptor currents after GABA and zinc co-application were plotted. (***p < 0.001 vs wt; ^††^p < 0.001 vs Q390*, ^§^p < 0.05 vs W429*). ANOVA with Bonferroni post hoc test was used.

Co-expression of the different mutant γ2 subunits resulted in different levels of surface expression of the wild-type partnering subunits. To confirm this we compared the peak current amplitude and zinc sensitivity of currents recorded from cells co-expressing α1 and β2 subunits with γ2, γ2(R136*), γ2(Q390*) or γ2(W429*) subunits. The peak currents from cells expressing the α1β2γ2(R136*) (724.8 ± 88.05, n = 10), α1β2γ2(Q390*) (214.4 ± 83.15, n = 8) or α1β2γ2(W429*) (1029 ± 95.48, n = 7) subunits were smaller than those recorded from cells co-expressing wild-type γ2 subunits (3502 ± 493.3, n = 6) (Fig. 8A). Compared to currents from cells co-expressing α1 and β2 subunits with wild-type γ2 subunits, currents recorded from cells co-expressing mutant γ2 subunits had enhanced zinc sensitivity, suggesting surface expression of α1β2 receptors with co-expression of all of the mutant subunits (Fig. 8A,C). Zinc (10 μM) application minimally reduced wild-type receptor currents (8.33 ± 1.29, n = 6) but reduced currents from cells co-expressing mutant γ2 subunits by ~80–90% (Fig. 8C).

## Discussion

We propose that differential protein structural disturbances in mutant GABA_A_ receptor γ2 subunits result in differential mutant γ2 subunit protein biogenesis, maturation, surface expression and ultimately total GABA-evoked current. The mutant γ2 subunits resulting from different mutations that produce different structural disturbances may be phenotype modifiers of their associated genetic epilepsies.

We have demonstrated that different mutant subunits are predicted to adopt different conformations. Consequently, these structurally altered mutant subunits had different protein surface hydrophobicities. The γ2(R136*) subunits only retained a short N-terminal upstream sequence, which were efficiently degraded inside cells. Based on the structure modeling and biological data, it is likely that γ2(R136*) subunits were not incorporated into the pentamer as the wild-type receptor. Therefore, the α1β2γ2(R136*) receptor current had a high sensitivity to zinc inhibition which suggests γ2 subunit was absent and the current was likely produced by α1β2 receptors. The γ2(Q390*) subunits adopted a new α-helix, became very aggregation-prone, were stable and inefficiently degraded while the γ2(W429*) subunits had a stability that was similar to wild-type γ2 subunits. Based on the buried surface area, mutant γ2(Q390*) subunit dimers had the highest energies while γ2(R136*) subunits had the lowest energies. Consequently, the γ2(R136*) subunit dimer was the least stable, the γ2(Q390*) subunit dimer was the most stable, and the γ2(W429*) subunit dimer had stability similar to wild-type γ2 subunit dimers. The γ2(R136*) subunit could not form heterodimers with binding partners like the α1 subunit while both γ2(Q390*) and γ2(W429*) subunits could form heterodimers with binding partners.

The mutant γ2(Q390*) subunit is the most stable protein and formed the most higher oligomers compared with γ2(R136*) and γ2(Q390*) subunits. Although both wild-type γ2 subunit and mutant γ2(Q390*) subunits could dimerize, the mutant γ2(Q390*) subunit formed the most higher oligomers compared to wild-type γ2 and mutant γ2(R136*) and γ2(W429*) subunits. Interestingly, although to a different degree, all mutant γ2 subunits were more likely to dimerize than wild-type γ2 subunits. It is likely that the hydrophobicity surface in the wild-type γ2 subunits that promote dimerization is somehow masked by co-assembly with other partnering subunits such as α1 and β2 subunits, while the hydrophobicity surface of mutant γ2 subunits could not be masked during subunit folding and assembly. Thus, mutant γ2 subunits are available to dimerize or to form the higher oligomers.

The interaction of γ2 subunits and wild-type partnering subunits like α1 or β2 subunits are different. The mutant γ2 subunits suppressed biogenesis of their partnering wild-type subunits. The docking study indicated that γ2(R136*) subunits had minimal interaction with wild-type α1 subunits. As the *ASA* located at the interface of proteins dominates their stability, γ2(R136*) subunits with only small remnant of the extracellular domain had minimal interaction with wild-type α1 subunits, consistent with the experimental biochemical observations. We demonstrated that the α1 subunit surface expression levels were unaltered when α1 subunits were co-expressed with β2 and γ2(R136*) subunits. In contrast, α1 subunit levels were most reduced when α1 subunits were co-expressed with γ2(Q390*) subunits and were reduced, but to a lesser extent, when co-expressed with γ2(W429*) subunits. The increased buried surface area or the high energies to form propagating dimers in γ2(Q390*) subunits may explain the strong dominant negative suppression of the partnering subunits like α1 subunits.

Reduced surface expression of mutant protein is a common observation among all GABA_A_ receptor subunit mutations^22^. The nonsense mutations in GABA_A_ receptor subunits results in loss of function of the subunit. We demonstrated that all of the mutant γ2 subunits had minimal surface expression, although the γ2(W429*) subunit had a small but significant increase of surface expression compared to the γ2(R136*) and γ2(Q390*) subunits. However, the significance of this small increase is unknown *in vivo* with a much crowded cellular environment and during development. As to the partnering α1 subunit, its surface expression was consistent with the total protein expression for each mutation. The surface expression of α1 subunits was unaltered when co-expressed with β2 and γ2(R136*) subunits but was reduced when co-expressed with β2 and γ2(Q390*) subunits or γ2(W429*) subunits. GABA_A_ receptors must traffick to the cell surface to conduct chloride ions. Those mutant subunits that are retained intracellularly are nonfunctional and may cause cellular toxicity like ER stress^4^.

Since only receptors trafficked to the cell surface are functional, and different mutant γ2 subunits result in differential surface expression of partnering subunits, we determined the total GABA-evoked current produced for receptors formed in the presence of each mutant γ2 subunit. When mutant γ2 subunits were co-expressed with α1 and β2 subunits, all of the currents were substantially reduced. However, the mutant α1β2γ2(Q390*) receptor channel current was the most reduced while the α1β2γ2(W429*) receptor current was the least reduced. With the zinc sensitivity test, it is likely that all the mutant currents were largely due to α1β2 receptor currents. This is consistent with the notion that β subunits compensate for γ subunits when they are lacking, and that the γ subunit is not essential for receptor assembly^23^ but is critical for receptor clustering at synapses^24^. In patients heterozygously harboring these *GABRG2* mutations, it is likely the mutant γ2 subunits are not present on the cell surface. Only the wild-type subunits will traffick to the cell surface and synapses.

In this study, homology modeling provides a promising method to obtain a high accuracy tertiary protein model, which could reveal substantial structural detail. This homology modelling can help to explain protein functions and molecular mechanisms. Once the structure was predicted, it could be treated as a monomer for docking predictions. In our study, the challenge in constructing dimers mostly comes from the membrane region, which restricts intracellular, transmembrane, and extracellular domains to bind accordingly to its counter part of the monomer. In this work, we filtered all the unqualified models in dimer construction. We used the structurally solved β3 homopentamer and hypothetical homopentamer structures to model GABA_A_ receptor subunits by aligning a monomer to corresponding position to the β3 template. Because our docking prediction of all of the wild-type and mutant GABA_A_ receptor subunits was template-based, the prediction is more accurate than with general docking.

In protein-protein interactions, many factors could influence the binding affinity, including hot spots, anchor residues, allosteric regulators and non-interface affinity modifiers. Hence, we used multiple quantitative criteria to infer the binding affinities of the protein complexes constructed from docking on structure prediction components. Compared with the experimental results, buried surface area based and empirical based methods are consistent with most biochemical and electrophysiological observations, while Rosetta based predictions succeed only in one observation. The limitation of structure based methods may come from Rosetta’s sensitive energy function, where small errors in structural conformation may produce large fluctuations in energy values.

In summary, as shown in Table 2, we demonstrated that different *GABRG2* mutations may result in mutant subunits with different protein conformations due to different structural disturbances and different functional consequences. This could be applied to other mutations associated with many human diseases. In this study, all the three *GABRG2* mutations (R136*, Q390*, W429*) resulted in a loss-of-function of the mutant subunits, which could not traffick to the cell surface and were retained inside ER with glycosylation arrest. However, the *GABRG2* R136* mutation resulted in a mutant subunit that had the least impact on partnering subunits due to its unstable binding with the partnering subunits, while the γ2(Q390*) subunits had the most dominant negative suppression of the wild-type partnering subunits due to the stable binding with partners during protein-protein interactions. The γ2(W429*) subunits had a mild dominant negative suppression on the wild-type partnering subunits. Therefore, the *GABRG2*(Q390*) mutation should result in a more severe phenotype compared with *GABRG2*(R136*) and *GABRG2*(Q390*) mutations.

## Methods

### Structural modeling of the wild-type and the mutant GABA_A_ receptor subunits

We mainly used our in-house protein structure prediction tool MUFOLD^6^ to construct protein models of GABA_A_ receptor α1, β2, β3, γ2, and δ subunits. We also carefully modeled mutant GABA_A_ receptor γ2 subunits including: (1) the γ2(R136*) subunit, with all transmembrane regions deleted and only part of the N-terminal domain remains; (2) the γ2(Q390*) subunit, with the fourth hydrophobic transmembrane α-helix (YARIFFPTAFCLFNLVYWVSYLYL) deleted and a new α-helix with many charged amino acids (KDKDKKKKNPAPTIDIRPRSATI) found to assume its location; and (3) the γ2(W429*) subunit, with the fourth hydrophobic transmembrane α-helix truncated. In the MUFOLD protocol, several experimental protein structures in PDB were identified based on homology as templates (PDB id: 4cof and 2bg9). Then multidimensional scaling (MDS) was used to reconstruct multiple protein decoys based on these templates, and these decoys were clustered and evaluated. With several iterations of model generation and evaluation, one decoy was chosen as the predicted protein model and then refined by Rosetta^25^. For mutant GABA_A_ receptor γ2 subunits, the original input subunits were split into different domains, and each domain was modeled individually and then assembled together.

To further understand the stability of the wild-type and mutant subunits, a dimer structure was constructed between two subunits in symmetric docking by SymmDock^26^. SymmDock used a priori restriction on its transformational search space only to symmetric transformations, which makes it gains both in efficiency and performance on cyclically symmetric homo-multimers. Because GABA_A_ receptor subunits are membrane proteins, special filtering on dimer models was applied to make sure the intracellular, transmembrane, and extracellular domains interacting correspondingly between the γ2 monomers. General docking was performed in conjunction with template-based docking^27^ between γ2 and α subunits by mapping their corresponding positions to the GABA_A_ receptor β3 homopentamer template (PDB id: 4cof). Heteropentamers and hypothetical homopentamers were also constructed by template-based docking. Chimera^28^ and Pymol^29^ were used to display the protein structural models.

### Quantitatively inferring stability of dimer and pentamer models

We used several quantitative methods to calculate buried surface area and force field to computationally infer the binding affinities of the proposed docking protein complexes (detailed in Supplementary Table 5). Buried accessible surface area (*ASA*) and buried hydrophobic accessible surface area (*hydroASA*) dominate binding affinity^30^, and we treat them as the *hydrophobicity score*. *ASA* is calculated as sum of the surface areas of two proteins monomers minus the surface of protein complex dimer. We used nACCESS^15^ software to get solvent *ASA* with solvent probe radius 1.4 Å. Between two proteins and the protein complex, buried surface area caused by carbon and sulfur atoms are defined as the Hydrophobic Buried Surface Area (*S*_*pho*_). buried surface area caused by oxygen and nitrogen atoms as the Hydrophilic Buried Surface Area (*S*_*phi*_). An empirical score (*EmpiricalValue*) was used to obtain the binding affinity by incorporating buried surface area and the hydrophobicity in empirical linear combination. *EmpiricalValue* incorporates buried hydrophobic surface area, and the weights came from previous work^31^ (Eq. (1). For a given protein complex, *EmpiricalValue* is calculated as (1):


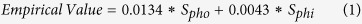


To incorporate the solvent characteristics in transmembrane domain and the extracellular/intracellular domains, we calculated *EmpiricalValue* from three individual domains:


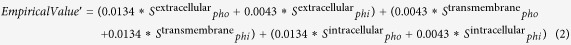


We also used *Choi’s dG_est,* which is another empirical based binding affinity calculation^32^. Its predicted binding energy *dG_est* value is obtained by estimating the contribution of the solvation factor in protein binding by a minimalistic solvation-based model. In addition, force field-based method Rosetta interface analyzer^33^,^34^,^35^ was applied to examine the quality and stability of protein-protein interaction interface. We chose the widely accepted binding energy per unit area (*dG_separated/dSASAx100*) and *packstat* (value from poor 0.0 to good 1.0) to illustrate the quality of the interface. In general, the value of *dG_separated/dSASAx100* below −1.5 and value of *packstat* above 0.65 are considered to be good. These two structure-based values are both from Rosetta interface analyzer of Rosetta Buddle 3.4.

### Expression vectors with GABA_A_ receptor subunits

The cDNAs encoding human α1, β2, and γ2 subunits in the pcDNA(3.1) vector with the cytomegalovirus (CMV) promoter were as described previously^36^,^37^. All the truncation mutations were generated using the QuikChange site-directed mutagenesis kit (Stratagene, La Jolla, CA) and confirmed by DNA sequencing in the Vanderbilt DNA Core. The short form of the γ2 subunit was used in this study, and numbering of γ2 subunit amino acids was based on the immature peptide that includes the 39 amino acids of the signal peptide.

### Cell culture and transfection

HEK 293T cells were replenished with DMEM supplemented with 10% FBS and 1% antibiotics. HEK 293-T cells for immunoblots were transfected with Fugene (Invitrogen, Carlsbad, CA). Cells were co-transfected with 1 μg of each subunit or 3 μg of single subunit plasmid for each 60 mm^2^ dish, and the total lysates were harvested 48 hr later.

### Western blot and protein digestion

Transfected HEK293T cells were collected in modified RIPA buffer (50 mM Tris (pH = 7.4), 150 mM NaCl, 1% NP-40, 0.2% sodium deoxycholate, 1 mM EDTA) and 1% protease inhibitor cocktail (Sigma). Collected samples were subjected to gel electrophoresis using 4–12% BisTris NuPAGE precast gels (Invitrogen) and transferred to PVDF-FL membranes (Millipore). Monoclonal anti-α1 subunit antibodies (NeuroMab) and polyclonal anti-γ2 subunit antibodies (Alomone or Millipore) were used to detect GABA_A_ receptor subunits. Anti-Na+/K + ATPase antibody (Abcam) was used as a loading control. IRDye® (LI-COR Biosciences) conjugated secondary antibody was used at a 1:10,000 dilution in all cases. Membranes were scanned using the Odyssey Infrared Imaging System (LI-COR Biosciences). The integrated intensity value of bands was determined using the Odyssey Image Studio software (LI-COR Biosciences).

For protein digestion, cell lysates were incubated with enzyme Endo H or PNGase F in G7 or G5 reaction buffer, respectively (New England BioLabs). Digestion proceeded for 3 h at 37 °C and was stopped with 5% β-mercaptoethanol (Sigma). Treated samples were then subjected to SDS-page electrophoresis and western blot.

### Measurement of surface GABA_A_ receptor subunit expression using flow cytometry

Measurement of surface expression of GABA_A_ receptor α1 and HA-tagged γ2 subunits using flow cytometry has been described previously^38^. Briefly, transfected HEK 293T cells were removed from the dishes by trypsinization and then resuspended in FACS buffer (phosphate buffered saline, PBS supplemented with 2% FBS and 0.05% sodium azide). Following washes with FACS buffer and permeabilization with Cytofix/cytoperm (BD Biosciences) for 15 min, cells were incubated with mouse monoclonal anti-HA antibody (1:200) or anti-α1 subunit antibody for 2 hours and then incubated with fluorophore Alexa-647 conjugated goat anti-mouse 2^nd^ antibody (1:2000) for 1 hour at 4 °C. Cells were then washed with FACS buffer and fixed with 2% paraformaldehyde. The acquired data were analyzed using FlowJo 7.1 (Treestar).

### Electrophysiology

HEK 293T cells were co-transfected with 2 μg of each subunit plasmid and 1 μg of the pHook-1 cDNA (Invitrogen, Carlsbad, CA) using a modified calcium phosphate precipitation method and selected 24 hours after transfection by magnetic hapten coated beads^39^. For each recording, the external bathing solution consisted of (in mM) NaCl 142, KCl 8, MgCl_2_ 6, CaCl_2_ 1, HEPES 10, glucose 10, pH 7.4 and 325–330 mOsm. The pipette solution consisted of (in mM) KCl 153, MgCl_2_ 1 MgATP 2, HEPES 10, EGTA 5, pH 7.3 and 310–320 mOsm. Recording pipettes were made of thin-walled borosilicate glass (World Precision Instruments, Pittsburgh, PA) pulled with a P-2000 laser puller (Sutter Instruments, San Rafael, CA) and fire polished with a microforge (Narishige, East Meadow, NY) to resistances between 1.2–1.8 MΩ when filled with internal solution. Lifted whole cells were voltage clamped at −50 mV^37^,^40^.

### Data analysis

Protein IDVs were quantified by using Odessy fluorescence imaging system (Li-Cor). Macroscopic currents were low pass filtered at 2 kHz, digitized at 10 kHz, and analyzed using the pClamp9 software suite (Axon Instruments, Union City, CA). Statistical significance of immunoblot flow cytometry and electrophysiology data was determined by ANOVA with Bonferroni posttests, a Student’s unpaired t test or, if appropriate, single-value t test (GraphPad Prism, La Jolla, CA). All analyses used an alpha level of 0.05 to determine statistical significance.

## Additional Information

**How to cite this article**: Wang, J. *et al*. Differential protein structural disturbances and suppression of assembly partners produced by nonsense GABRG2 epilepsy mutations: implications for disease phenotypic heterogeneity. *Sci. Rep.* **6**, 35294; doi: 10.1038/srep35294 (2016).

## Supplementary Material

Supplementary Information

## Figures and Tables

**Table 1:** Summary of the binding affinity of GABRG2 mutant subunits.

Index	Binding Affinity of homo-dimer	Binding Affinity of pentamer α-β-α-β-γ2 pentamer
Wide-type γ2	stable	stable
Mutant γ2(R136*)	least stable	least stable
Mutant γ2(Q390*)	most stable	most stable
Mutant γ2(W429*)	same as wild-type	same as wild-type

**Table 2:** The structural disturbances and molecular defects of GABRG2 nonsense mutations.

	R136*	Q390*	W429*
Dimer hydrophobicity	low	high	moderate
Homodimer	yes	yes	yes
Heterodimer	no	yes	yes
Higher oligomer	yes	yes	yes
Surface expression	no	no	No
Suppression of Binding partners	no	yes	yes/moderate
Glycosylation arrest	yes	yes	yes
Channel function	reduced	reduced	reduced
