# Supplementary material for: Differential protein structural disturbances and suppression of assembly partners produced by nonsense GABRG2 epilepsy mutations: implications for disease phenotypic heterogeneity
Source: Sci Rep. 2016 Oct 20;6:35294. doi: 10.1038/srep35294 (PMC5071880; doi:10.1038/srep35294)

DIFFERENTIAL PROTEIN STRUCTURAL DISTURBANCES AND SUPPRESSION OF ASSEMBLY PARTNERS PRODUCED BY NONSENSE GABRG2 EPILEPSY MUTATIONS: IMPLICATIONS FOR DISEASE PHENOTYPIC HETEROGENEITY

Juexin Wang PhD[1*], Dingding Shen[2*] ,MD, Geqing Xia[2], MD,PhD, Wangzhen Shen[2], MD, Robert L. Macdonald MD, PhD[1, 2, 3, 5], Dong Xu[1], PhD, Jing-Qiong Kang MD, PhD[2, 5]

[1]Department of Computer Science and Christopher S. Bond Life Sciences Center, University of Missouri, Columbia, MO, 65211

[2]Departments of Neurology, Molecular Physiology and Biophysics[2] and Pharmacology[3], The Graduate Program of Neuroscience[4], and the Vanderbilt Brain Institute[5]

Vanderbilt University Medical Center

Nashville, TN 37212

Supplementary Table 1. Binding affinity quantitative criteria on different mutant γ2 subunits form homo-dimers with different stabilities

Name	Buried Surface Area	Hydrophobic Buried Surface ASA	EmpiricalValue	EmpricialValue'	Choi's dG_est	dG_separated/dSASAx100	Packstat
Wide-type γ2	2342.576	1675.349	25.319	NA	-13.26	104.139	0.464
Mutant γ2(R136*)	2513.002	1556.015	24.966	NA	-9.09	474.118	0.558
Mutant γ2(Q390*)	2475.417	1796.585	26.993	NA	-9.13	233.474	0.482
Mutant γ2(W429*)	2697.394	1679.967	26.886	NA	-9.7	17.501	0.423

Supplementary Table 2. Binding affinity quantitative criteria on wide-type GABAR subunits

Name	Buried Surface Area	Hydrophobic Buried Surface ASA	EmpiricalValue	EmpricialValue'	Choi's dG_est	dG_separated/dSASAx100	Packstat
GABAAR-α	2247.564	1372.385	22.153	NA	-11.12	341.663	0.461
GABAAR-β2	2358.566	1558.47	24.324	NA	-12.48	456.072	0.534
GABAAR-β3	2300.28	1487.457	23.4277	NA	-10.39	278.083	0.645
GABAAR-δ	2150.594	1214.823	20.302	NA	-10.03	129.653	0.575
GABAAR-γ2	2342.576	1675.349	25.319	NA	-13.26	104.139	0.464

Supplementary Table 3. Binding affinity quantitative criteria on interaction of the mutant γ2 subunits with the wild-type partnering subunits

Name	Buried Surface Area	Hydrophobic Buried Surface ASA	EmpiricalValue	EmpiricalValue'	Choi's dG_est	dG_separated/dSASAx100	Packstat
pentamer α-β-α-β-γ2(R136*)	1123.039	654.1	10.781	10.781	-9.37	351.0895	0.508
pentamer α-β-α-β-γ2(Q390*)	4725.071	3229.327	49.705	31.983	-12.76	326.311	0.516
pentamer α-β-α-β-γ2(W429*)	4120.845	2734.375	42.602	33.753	-12.41	315.1405	0.5805
pentamer α-β-α-β-γ2	4149.771	2744.97	42.823	46.491	-12.41	310.7	0.5445

Supplementary Table 4. Details in interface binding affinities by quantitative criteria on interaction of the mutant γ2 subunits with the wild-type partnering subunits

Name	Buried Surface Area	Hydrophobic Buried Surface ASA	EmpiricalValue	EmpiricalValue'	Choi's dG_est	dG_separated/dSASAx100	Packstat
γ2(R136*)_α	487.011	268.335	4.536	4.5359958	-9.86	0.43	0.272
γ2(R136*)_β3	1759.066	1039.865	17.0268	17.0267553	-8.88	701.749	0.744
γ2(Q390*)_α	4729.686	3315.521	50.5089	27.1772649	-13.22	477.717	0.503
γ2(Q390*)_α	2642.559	1586.729	25.802		-10.7	527.193	0.564
γ2(Q390*)_α	257.206	227.641	1.3753		-8.47	8.585	0.462
γ2(Q390*)_β3	4720.455	3143.133	48.900	36.7894956	-12.3	174.905	0.529
γ2(Q390*)_β3_p1	2857.863	1696.21	27.724		-10.49	144.395	0.634
γ2(Q390*)_β3_p2	1344.309	983.359	9.0652		-9.61	96.252	0.494
γ2(W429*)_α	5063.039	3507.032	53.685	36.4813397	-13.48	484.282	0.494
γ2(W429*)_α_p1	2598.362	1571.929	25.478		-10.7	519.845	0.486
γ2(W429*)_α_p2	1735.684	1403.024	10.491		-9.6	463.565	0.418
γ2(W429*)_α_p3	43.366	35.902	0.513		-8.47	0	NA
γ2(W429*)_β3	3178.651	1961.717	31.520	31.0254521	-11.34	145.999	0.667
γ2(W429*)_β3_p1	2812.69	1656.06	27.165		-10.48	135.046	0.611
γ2(W429*)_β3_p2	328.803	263.077	2.0120		-8.93	97.793	0.471
γ2(W429*)_β3_p3	271.486	74.878	1.849		-8.47	NA	NA
γ2_α1	5134.923	3535.588	54.254	36.912574	-13.48	475.399	0.467
γ2_α1_p1	2598.364	1571.923	25.477		-10.7	519.845	0.533
γ2_α1_p2	1720.879	1393.051	10.383		-9.6	463.332	0.452
γ2_α1_p3	111.936	62.722	1.052		-8.47	0.466	0.355
γ2_β3	3164.619	1954.351	31.392	56.0689895	-11.34	146.001	0.622
γ2_β3_p1	2812.692	1656.054	27.165		-10.48	135.046	0.619
γ2_β3_p2	313.997	253.103	1.904		-8.93	97.792	0.462
γ2_β3_p3	272.842	76.138	1.866		-8.47	Infinity	NA

P1,P2,P3 are different domains of the dimer. P1 is extracellular part, P2 is transmembrane part and P3 is intracellular part.

Supplementary Table 5. Assessments criteria of protein-protein binding affinity

No	Name	Category	Absolute Value/ Relative Value	Short Description	Tool	Correlate with stability
1	Buried Surface Area	Buried Area	Absolute Value	(Solvent) Accessible Surface Area as the difference between surface area of the complex and the sum of the surface areas of the two proteins	nACCESS	Positive
2	Hydrophobic Buried Surface ASA	Buried Area	Absolute Value	ASA Buried Area caused by Carbon and Sulfur atoms	nACCESS	Positive
3	EmpiricalValue	Empirical Score	Relative Value	Linear combination of Empirical weights on Hydrophobic ASA and Hydrophilic ASA	nACCESS	Positive
4	EmpiricalValue	Empirical Score	Relative Value	Different weights on Hydrophobic ASA and Hydrophilic ASA of transmembrane and extracellular/intracellular domains	nACCESS	Positive
5	Choi's dG_est	Empirical Score	Absolute Value	Linear combination of buried surface areas according to amino-acid types	minipredictor	Positive
6	dG_separated/dSASAx100	Force Field	Absolute Value	Binding energy per unit area, the dG_separated binding energy divided by the total interface surface area	Rosetta Interface Analyzer	Negative
7	Analyzer's Packstat	Force Field	Absolute Value	How well packed the interface is with 0.0 being as poor as possible and 1.0 being perfect shape complementarity	Rosetta Interface Analyzer	Positive

Seven criteria to quantitatively infer the binding affinity and they can be grouped into three categories. The calculated value can be either absolute or relative and they are positively or negatively correlated with the stability.

Supplementary Figure 1

Full-length gels for Figure 5B and Figure 7A

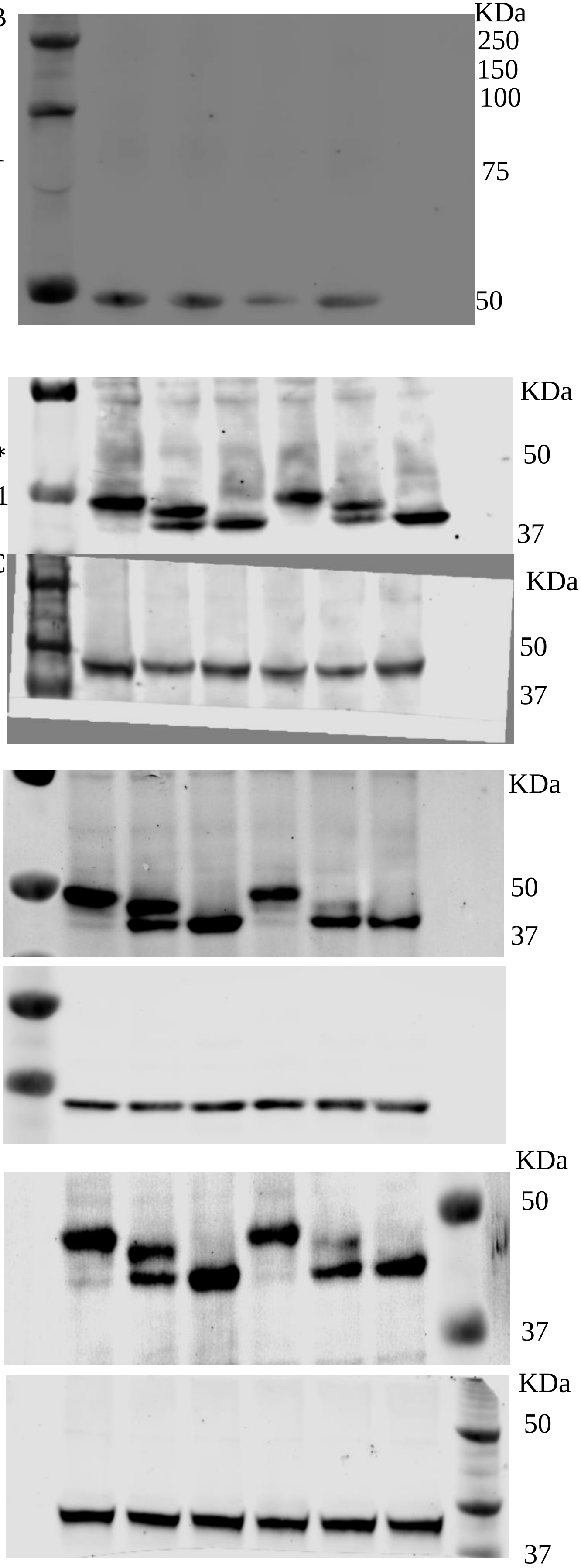

Supplement: Supplementary Information [file srep35294-s1.pdf]
